# ‘Stand still …, and move on’ , an early neurologically-focused follow-up for cardiac arrest survivors and their caregivers: a process evaluation

**DOI:** 10.1186/1472-6963-14-34

**Published:** 2014-01-23

**Authors:** Véronique RM Moulaert, Jolanda CM van Haastregt, Derick T Wade, Caroline M van Heugten, Jeanine A Verbunt

**Affiliations:** 1 Adelante, Centre of Expertise in Rehabilitation and Audiology, Zandbergsweg 111, 6432 CC Hoensbroek, the Netherlands; 2 Department of Rehabilitation Medicine, CAPHRI School for Public Health and Primary Care, Maastricht University Medical Centre+, Maastricht, the Netherlands; 3 Department of Health Services Research, CAPHRI School for Public Health and Primary Care, Maastricht University Medical Centre+, Maastricht, the Netherlands; 4 Oxford Centre for Enablement, Nuffield Orthopaedic Centre, Oxford, UK; 5 School for Mental Health and Neuroscience, Maastricht University, Maastricht, the Netherlands; 6 Department of Neuropsychology and Psychopharmacology, Maastricht University, Maastricht, the Netherlands

**Keywords:** Cardiac arrest, Hypoxic-ischaemic brain injury, Cognitive impairments, Emotional impairments, Caregiver, Nursing intervention, Feasibility, Process evaluation

## Abstract

**Background:**

A cardiac arrest can lead to hypoxic-ischaemic brain injury which can result in cognitive and emotional impairments and may negatively affect daily functioning, participation in society and quality of life. Furthermore, the impact on the family of the patient can be high. We designed an intervention called ‘Stand still …, and move on’, which is a concise, individualised, semi-structured intervention for survivors of cardiac arrest and their caregivers, consisting of between one and six face-to-face consultations provided by a trained nurse. The intervention is directed at early detection of cognitive and emotional problems, provision of information, promotion of self-management and referral to specialised care if necessary. The effectiveness of the intervention is being examined in a randomised controlled trial [ISRCTN74835019]. Alongside this trial we performed a process evaluation which aims to investigate the feasibility of the intervention by assessing: 1) the attendance and dose delivered; 2) performance according to protocol; and 3) the opinion of patients, caregivers and nurses on the intervention.

**Methods:**

Participants of this process evaluation were 97 patients allocated to the intervention group of the RCT, their 91 caregivers, and six nurses who conducted the intervention. Measurement instruments used were evaluation forms for patients and caregivers, registration and evaluation forms for nurses, and semi-structured interviews with nurses.

**Results:**

Seventy-nine of the patients (81%) allocated to the intervention group and 65 caregivers (71%) participated in the intervention. The mean (SD) number of consultations per patient was 1.8 (1.0), and most consultations were conducted at the patients’ home. The intervention was performed largely according to protocol, except that the intervention usually started later than intended, consultations were longer than expected, and the topic of self-management was not regularly addressed. Patients marked the quality of the intervention with a mean score of 7.5 and the performance of the nurse with an 8.0 out of ten. Overall, the intervention was positively evaluated by patients, caregivers and nurses.

**Conclusions:**

The intervention ‘Stand still …, and move on’ is a promising intervention which was performed largely according to protocol and seems feasible for implementation after some adaptations, if it is found to be effective.

## Background

Surviving a cardiac arrest is a major life event. Persons who survive a cardiac arrest may suffer from hypoxic-ischaemic brain injury due to the temporary cessation of blood circulation in the brain [1]. This can lead to cognitive and emotional impairments, and may affect daily functioning, participation in society and quality of life [2-4]. Furthermore, a cardiac arrest can have considerable impact on the family and caregivers in terms of psychosocial problems and high perceived care burden [4].

In literature only a few aftercare interventions for survivors of cardiac arrest have been described [5]. We identified two psychosocial interventions that resulted in improved knowledge and reduced physical and emotional symptoms [6,7]. Although these interventions addressed psychosocial topics, they did not include screening for possible cognitive impairments. We have previously shown that cognitive impairments occur in almost fifty percent of the survivors of cardiac arrest and are related to a reduced quality of life, which suggests that early detection of cognitive impairments may be important [8,9]. We therefore developed an early intervention service called ‘Stand still …, and move on’ , which is directed at early detection of cognitive and emotional problems, provision of information on possible consequences of a cardiac arrest, promotion of self-management and referral to specialised care if necessary [5]. The main goal of this intervention was to improve societal participation and quality of life, and the effect of this intervention is examined in a randomised controlled trial (RCT): ‘Activity and Life After Survival of a Cardiac Arrest’ (ALASCA) [ISRCTN74835019] [10]. In this RCT the intervention group received the new intervention, while the control group received care as usual.

In the current paper we present the results of the process evaluation of this intervention, which was performed alongside the trial. A process evaluation is a systematic way to monitor the delivery of an intervention and can provide insight into factors that may have influenced the effectiveness of the intervention and can help to understand why an intervention was effective or not [11]. We performed this process evaluation prior to analysing the results of the trial, in order to prevent possible bias related to already knowing the effectiveness of the intervention [12].

The aim of this process evaluation was to evaluate the feasibility of the intervention by assessing: 1) the attendance and dose delivered; 2) performance according to protocol; and 3) the opinion of patients, caregivers and nurses on the intervention.

## Methods

### Intervention

The intervention ‘Stand still …, and move on’ is a concise, individualised, semi-structured intervention, for survivors of cardiac arrest and their caregivers [5]. The intervention is conducted by trained nurses and consists of between one and six face-to-face consultations, depending on the individual needs of patient and caregiver. The first consultation is planned soon after discharge from hospital, preferably within one month. Both patient and caregiver are invited to the consultations. If the patient has a caregiver who decides to participate, patient and caregiver attend the consultations together. The first consultation has an intended duration of approximately 60 minutes and follow-up sessions are intended to last about 30 minutes. The consultations take place in hospital or at the patients’ home. Additional consultation by telephone is possible.

The intervention is designed for the early detection of cognitive and emotional consequences of cardiac arrest and consists of four elements: 1) screening for cognitive and emotional problems; 2) provision of information and support; 3) promotion of self-management strategies; and 4) referral to specialised care if indicated. Below, each element will be described in more detail.

First, the nurse screens for signs of cognitive impairments in the patient and for possible emotional problems in patient and caregiver by conversation and observation. In addition, the nurse can use one of the following screening instruments: Checklist Cognition and Emotion (CLCE-24) [13], Cognitive Log (Cog-log) [14], Hospital Anxiety and Depression Scale [15], Impact of Event Scale [16] and the Caregiver Strain Index [17].

Second, the nurse provides information about possible consequences of cardiac arrest. She also hands out an information booklet that was developed for this intervention. In this information booklet frequently occurring cognitive and emotional problems after cardiac arrest are described, and suggestions for effective coping strategies are provided. In addition, the nurse has a set of brochures from patient organisations on topics such as medication, cardiac treatments, memory problems, fatigue or driving restrictions, which she can offer at indication.

Third, self-management strategies are promoted. The goal of self-management is to stimulate patients to take responsibility for actions that should give the best quality of life given their possibilities [18]. Self-management interventions have shown to be effective in several chronic illnesses, including in heart failure [19,20]. To promote self-management, self-management skills such as problem solving and making action plans can be practiced with the participants if needed [19].

Finally, the nurse discusses whether referral to specialised care is indicated. Referrals can be made, for example, to a cardiologist in case of cardiac symptoms or concerns, to a neuropsychologist for further cognitive testing and treatment, or to a consultant in rehabilitation medicine to evaluate the need for multi-disciplinary rehabilitation treatment. More details on the rationale and description of the intervention have been published elsewhere [5].

### Participants

#### Patients

The 97 patients eligible for this process evaluation were all survivors of a cardiac arrest who were allocated to the intervention group of the ALASCA trial. Inclusion for the ALASCA trial took place between April 2007 and November 2010. During this period 185 patients were included for the trial at the coronary care units and intensive care units of five hospitals in the Netherlands. Inclusion criteria for the ALASCA study were: survival more than two weeks after in-hospital or out-of-hospital cardiac arrest, living within 50 km of one of the participating hospitals, age 18 years or older and sufficient knowledge of the Dutch language. Exclusion criteria were a life expectancy lower than 3 months (as evaluated by the treating physician) and living in residential or institutional care prior to the cardiac arrest.

#### Caregivers

Ninety-one of the 97 patients in the intervention group had a caregiver. Caregiver was defined as partner, spouse, or other informal caregiver closely related to the patient. There were no additional in- or exclusion criteria for the caregivers.

#### Nurses

Six nurses undertook the intervention. Prior to the start of the interventions, training was offered to the nurses directed at acquiring skills to detect cognitive and emotional problems and promoting self-management among survivors of cardiac arrest. In addition, once a year a booster session was organised, and throughout the intervention period the nurses could contact a consultant in rehabilitation medicine (JV) for advice.

### Data collection

Table 1 describes the measurement instruments used in this process evaluation. All patients allocated to the intervention group, who had participated in at least one consultation, received an evaluation form after the last consultation. Also all caregivers who had been present in at least one of the consultations received an evaluation form. Evaluation forms were sent by mail and non-responders received one reminder. The nurses registered the course and contents of the intervention after each consultation on a registration form, and after the last consultation of each patient the nurses filled out an evaluation form. Finally, after completion of the trial the nurses were invited for interviews.

**Table 1:** Outcome measures and measurement instruments of the process evaluation

	**Evaluation form patient**	**Evaluation form caregiver**	**Registration form nurse**	**Evaluation form nurse**	**Interview nurses**
**1. Attendance and dose delivered**					
- Attendance patients and caregivers			x		
- Frequency and duration consultations			x		
**2. Performance according to protocol**					
- Characteristics consultations			x		
- Course and contents consultations	x	x	x	x	x
**3. Opinion on the intervention**					
- Opinion patients	x				
- Opinion caregivers		x			
- Opinion nurses				x	x

#### Evaluation form patient

The evaluation form for the patient consisted of thirteen multiple choice questions and statements regarding the course, contents and quality of the intervention. In addition, patients were asked to give a grade (10-point scale, ranging from 1 to 10) for the perceived quality of the intervention and the performance of the nurse. In three open questions the patients were asked to mention strong and weak points of the intervention and to provide suggestions for improvements.

#### Evaluation form caregiver

The evaluation form for the caregiver was the same as the evaluation form for the patients, except that questions were formulated from the perspective of the caregiver.

#### Registration form nurse

The registration form for the nurses included questions concerning characteristics of the intervention, including frequency, duration, start and location of the consultations, and the presence of the caregiver. Furthermore, the nurses registered the course and contents of the intervention, including the topics discussed, use of screening instruments, delivery of the information booklets and the extent to which self-management techniques had been practised with the patients.

#### Evaluation form nurse

After the last consultation with each patient, the nurses filled out an evaluation form on which they registered their perceived usefulness of the intervention for patient and caregiver. In addition, there were three open questions concerning strong and weak points of the intervention and suggestions for improvements.

#### Interview nurses

After completion of the trial, the two nurses that had conducted most interventions were invited to participate in an individual semi-structured interview by a researcher who had not been involved in the intervention process (JvH). During this interview, the nurses were invited to evaluate the intervention. In addition, two other nurses were invited to participate in a more concise telephone interview by the principal researcher (VM). The remaining two nurses were not approached for an interview as they both had stopped working on the project prior to the end of the trial, and one of the nurses had seen one patient only.

### Data analysis

The quantitative data from the registration and evaluation forms were analysed with descriptive statistics using SPSS, version 20. Qualitative data, resulting from the open questions on the evaluation forms and the interviews with the nurses, was classified into categories based on the contents of the answers.

### Ethical considerations

The Medical Ethics Committee of the University Hospital Maastricht/Maastricht University approved the ALASCA study. The study is registered in a public trial registry [ISRCTN74835019]. Patients and caregivers participating in this process evaluation had signed an informed consent form at the start of the ALASCA study.

## Results

### Characteristics participants

The mean age of the 97 patients allocated to the intervention group was 60 years (SD 12) at the moment of their cardiac arrest and 80 (82%) were male. The majority of the cardiac arrests (n = 77, 79%) had occurred outside the hospital.

The caregivers of the patients had a mean age of 57 years (SD 11) and 77 (88%) were female. Caregivers were spouses/partners (n = 82, 92%), children (n = 4, 5%) or other family members (n = 3, 3%).

The six nurses were all women with an age ranging from 40 to 57 years. They were experienced nurses who had been working for more than 15 years in the field of neurology (n = 2), cardiology (n = 3) or intensive care medicine (n = 1). Three nurses followed a 12-hour group training and three nurses that started later received a more compact and personal training.

### Attendance patients and caregivers

Of the 97 patients allocated to the intervention group, 79 patients (81%) actually received the intervention. Ten patients (10%) did not receive the intervention because they stopped their participation in the ALASCA study prior to the start of the intervention because of death (n = 2), medical problems (n = 3), high burden/lack of time (n = 3) or lack of interest (n = 2). Six persons (6%) refused the intervention while they continued their participation in the ALASCA study. Reasons for refusal were medical problems (n = 1), being already in rehabilitation treatment (n = 2) or lack of interest (n = 3). Furthermore, one patient did not receive the intervention due to logistical problems and for one person the reason was unknown.

Ninety-one of the 97 patients allocated to the intervention group had a caregiver. Of the 79 patients who actually received the intervention, 75 patients had a caregiver. Ten of these caregivers did not participate in the intervention, because of divorce (n = 2) or because they could not be present at the moment of the consultation (n = 8). Overall, 65 (71%) of the 91 caregivers have received the intervention.

### Response

The patient evaluation form was returned by 58 (73%) of the 79 patients who had received the intervention. The caregiver evaluation form was returned by 49 (75%) of the 65 caregivers who had participated in the intervention. The nurses filled out 136 registration forms (96%) about the 141 consultations they had conducted, and we received 75 evaluation forms (94%). The two nurses who were invited for the semi-structured interview and the two nurses who were invited for the telephone interview, all agreed to participate in the interviews.

### Frequency and duration consultations

Patients that participated in the intervention received a mean number of 1.8 consultations (SD 1.0, range 1 – 5). The majority of patients (n = 41, 52%) received one consultation. The duration of the face-to-face consultations did not change over time, and was as follows: six consultations (5%) were shorter than 30 minutes, a quarter (n = 30) lasted 30 to 60 minutes, two thirds (n = 81) had a duration of 60 to 90 minutes, and 4 consultations (3%) lasted more than 90 minutes. The telephone consultations had a mean duration of 16 minutes (SD 8, range 5 – 30). Nurses spent on average 18 minutes on preparation and administration per consultation.

### Characteristics consultations

On average the first consultation was conducted 90 days after the cardiac arrest (SD 59, range 19 – 344). Consultations were most frequently performed at the patients’ home (n = 80, 68%). The remaining consultations were performed in hospital, of which 25 (21%) at an outpatient clinic and thirteen (11%) at a clinical ward. Of the 141 consultations, nineteen consultations (13%) were conducted by telephone. Three patients received an intervention that consisted of telephone consultations only.

### Course and contents consultations

The nurses registered whether the four elements of the intervention had been addressed during the consultations.

#### Screening for cognitive and emotional problems

The topics ‘cognition’ and ‘emotion’ were discussed or addressed in at least one of the consultations in 62 (83%) and 61 (82%) patients respectively. In addition, the nurses used the following screening instruments during the consultations: Checklist Cognition and Emotion (n = 32), Hospital Anxiety and Depression Scale (n = 2), Impact of Event Scale (n = 20) and Caregiver Strain Index (n = 22).

#### Provision of information and support

Table 2 shows the topics that have been discussed during the consultations. Topics that were addressed most frequently were: daily activities, cognitive changes, emotional changes, physical changes, caregiver strain and fatigue.

**Table 2:** Number of patients with whom a topic was discussed during the intervention (n = 75)

**Topic**	**n**	**(%)**
Daily activities	67	(89%)
Cognitive changes	62	(83%)
Emotional changes	61	(81%)
Physical changes	56	(75%)
Caregiver strain	55	(73%)
Fatigue	53	(71%)
Driving	43	(57%)
Family and children	40	(53%)
Participation in society (including work)	40	(53%)
Behavioural changes	38	(51%)
Cardiologic questions	35	(47%)
Contacts with friends	26	(35%)
Implantable cardioverter defibrillator	15	(20%)
Partner relationships and sexuality	15	(20%)
Self-management	14	(19%)
Dealing with health care providers	12	(16%)

The information booklet was offered to 68 of the patients (92%). Of the participants who had received the booklet, 47 patients (83%) and 41 caregivers (87%) reported that they had read it. In addition, the nurses presented 26 other brochures to 19 patients on the following topics: medication for heart diseases (n = 13), implantable cardioverter defibrillator (n = 3), percutaneous coronary intervention (n = 3), myocardial infarction (n = 2), fatigue (n = 2), driving restrictions (n = 2), smoking (n = 2), heart failure (n = 1), sport (n = 1) and information about the hospital (n = 1).

#### Promotion of self-management strategies

The topic of self-management was addressed during at least one of the consultations in 14 patients (19%), and self-management techniques were practiced with 4 patients (5%).

#### Referral to specialised care

During the intervention, 13 patients (18%) were referred to specialised care. Patients were referred to a consultant in rehabilitation medicine (n = 7), psychologist (n = 2), neurologist (n = 1), social worker (n = 1), general practitioner (n = 1) and physiotherapist (n = 1).

### Opinion patients and caregivers

Table 3 shows that most patients and caregivers found the intervention useful and reported that they received enough information, advice and support. The quality and timing of the information booklet were evaluated positively, although eight patients (16%) and eight caregivers (19%) would have preferred to receive the booklet earlier. After the intervention, most patients and caregivers felt capable of dealing with the consequences of the cardiac arrest and stated that they would recommend the intervention to others.

**Table 3:** Opinion of patients and caregivers on the intervention

		**Patient**		**Caregiver**	
**Statement**	**Response**	**n**	**%**	**n**	**%**
‘The intervention was useful for me’	Yes	30	(54%)	27	(56%)
	Somewhat	17	(30%)	14	(29%)
	No	6	(11%)	6	(13%)
	No opinion	3	(5%)	1	(2%)
‘The intervention was useful for my partner/caregiver’	Yes	32	(59%)	30	(63%)
	Somewhat	13	(24%)	13	(27%)
	No	6	(11%)	4	(8%)
	No opinion	3	(6%)	1	(2%)
‘Problems were recognised’	Yes	32	(59%)	32	(71%)
	No	3	(6%)	3	(7%)
	No opinion	19	(35%)	10	(22%)
‘I received enough information about possible consequences of a cardiac arrest’	Yes	49	(86%)	43	(92%)
	No	4	(7%)	1	(2%)
	No opinion	4	(7%)	3	(6%)
‘I received enough practical tips and advices’	Yes	47	(84%)	38	(83%)*
	No	4	(7%)	4	(9%)
	No opinion	5	(9%)	4	(9%)
‘I felt sufficiently supported by the nurse’	Yes	46	(82%)	38	(81%)
	No	4	(7%)	3	(6%)
	No opinion	6	(11%)	6	(13%)
‘The quality of the information booklet was …’	(very) good	40	(85%)	35	(85%)
	Reasonable	7	(15%)	6	(15%)
	(very) poor	0	(0%)	0	(0%)
‘The timing of the information booklet was …’	Just right	39	(78%)	29	(71%)
	Too early	3	(6%)	4	(10%)
	Too late	8	(16%)	8	(19%)
‘I feel capable now of dealing with the consequences of the cardiac arrest’	Yes	48	(89%)*	38	(86%)
	No	3	(6%)	2	(5%)
	No opinion	3	(6%)	4	(9%)
‘I would recommend the intervention to others’	Yes	47	(85%)	42	(89%)
	No	2	(4%)	1	(2%)
	No opinion	6	(11%)	4	(9%)

*Percentages have been rounded off to whole numbers and therefore do not always add up to hundred percent.

Patients graded the perceived quality of the intervention with a mean score of 7.5 (SD 1.7, range 1 – 10) and caregivers gave a 7.4 (SD 1.6, range 3 – 10). Both patients and caregivers marked the performance of the nurse with an 8.0 (SD 1.1, range 3 – 10). Patients and caregivers that had received more than one consultation reported, on average, higher scores for the quality of the intervention and the performance of the nurse.

Strong aspects of the intervention, as reported by the patients and caregivers, were the opportunity to ask questions, the information provided and, most of all, the personal attention of the nurses, not only for the patient, but also for the caregiver. The most frequently reported weakness was the late start of intervention. A suggestion made by several participants was to add a group session with other patients who survived a cardiac arrest and their caregivers.

### Opinion nurses

The nurses considered the intervention useful for most patients (n = 71, 95%) and also for most caregivers (n = 61, 95%). All nurses recommended implementation of the intervention into regular health care. The interviews showed that nurses had different opinions on the optimal number of consultations per patient. Two nurses regarded one consultation sufficient for most patients, while the other two nurses considered it important to see patient and caregiver more frequently. All nurses reported that face-to-face contact was essential and could not be replaced by telephone calls. According to the nurses, home visits were highly appreciated by the patients, but were more time consuming for them due to travel time. The three nurses who had used the Checklist Cognition and Emotion, considered it to be a valuable screening instrument, mainly because it helped to structure the conversation. All nurses mentioned that the information booklet provided useful information for patients and caregivers, which is not present in regular brochures. During the interviews, the nurses explained that they regarded self-management not particularly useful for this patient group on this moment after their cardiac arrest. They considered self-management more suitable for more extensive interventions provided during the chronic phase.

The main strong aspects of the intervention reported by the nurses were the time, attention and open conversations they had with patients and caregivers. In addition, they valued that the well-being and burden of the caregiver was specifically addressed. The nurses reported that the information they could provide was highly appreciated. According to the nurses, the protocol provided sufficient structure to conduct the intervention and was feasible in most cases. Also, the semi-structured format of the intervention was positively evaluated as this enabled them to tailor the intervention to the individual needs and wishes of patient and caregiver.

The nurses also reported some weaknesses. Their main comment was that the intervention started too late. Besides, the nurses considered the intervention to be less useful for two groups of patients, namely for patients who experienced no problems at all and for patients that had already started a rehabilitation treatment. A suggestion made by one nurse was to introduce the intervention to patient and family already during hospital admission. As such, patients and family know what they can expect and rely on after discharge from hospital.

### Differences between nurses

Table 4 shows how the six nurses performed several aspects of the intervention. Differences can be noticed concerning number of consultations they conducted per patient, use of screening instruments and referral to specialised care. Next to that, Table 4 also shows that the scores given by patients and caregivers for perceived quality of intervention and performance of the nurse varied between the nurses.

**Table 4:** Differences in performance of the intervention across the nurses

	**Nurse A**	**Nurse B**	**Nurse C**	**Nurse D**	**Nurse E**	**Nurse F**
**Number of patients**	28	25	14	7	4	1
**Consultations per patient**						
*mean (SD)*	2.4 (1.1)	1.2 (0.6)	1.4 (0.6)	2.3 (1.0)	1.8 (0.5)	1.0 (n.a.)
**Screening instruments**						
- CLCE-24	23	0	0	6	3	0
- Cognitive Log	0	0	0	0	0	0
- HADS	0	0	2	0	0	0
- IES	19	0	0	0	0	0
- CSI	20	0	1	0	0	0
**Patients referred to specialised care**	6	5	1	1	0	0
**Quality intervention**						
*Grades mean (SD)*						
- According to patient	8.0 (1.1)	7.2 (2.0)	7.0 (1.9)	8.0 (1.4)	6.0 (2.0)	9.0 (n.a.)
- According to caregiver	7.8 (1.0)	7.0 (2.2)	6.7 (1.9)	7.6 (1.5)	8.0 (n.a.)	8.0 (n.a.)
**Performance nurse**						
*Grades mean (SD)*						
- According to patient	8.3 (0.8)	8.0 (1.1)	7.1 (2.0)	7.7 (1.1)	7.7 (0.6)	9.0 (n.a.)
- According to caregiver	8.2 (0.9)	8.2 (1.2)	7.0 (2.1)	8.3 (0.5)	8.0 (n.a.)	8.0 (n.a.)

CLCE-24 = Checklist Cognition and Emotion.

HADS = Hospital Anxiety and Depression Scale.

IES = Impact of Event Scale.

CSI = Caregiver Strain Index.

n.a. = not applicable.

## Discussion

We have studied the feasibility of an intervention by nurses for people who have survived a cardiac arrest and we found that most patients and caregivers participated and received on average 1.8 consultations. Nurses followed the protocol in most aspects, but the intervention started later after the event than intended, consultations were longer than expected, and self-management was rarely discussed. Overall, the intervention was positively evaluated by patients, caregivers and nurses.

The late start of the intervention, on average 90 days after cardiac arrest instead of the intended 1 month after discharge, can be mainly contributed to the design and organisational aspects of the ALASCA trial as, prior to randomisation, patients had to be informed about the trial, give their consent and perform the first measurements for the study. One can question whether the delayed start of the intervention may have influenced its effectiveness. We think that performing the first consultation earlier, that is more according to protocol, will probably increase the effectiveness because potential problems are addressed earlier, which may prevent some of the future negative consequences. Also the evaluations from patients, caregivers and nurses have shown that it is important to reduce this delay, which seems feasible in case of implementation of this intervention outside a scientific trial.

The duration of most consultations was longer than intended, namely 60 – 90 minutes, instead of the intended 60 minutes for first consultations and 30 minutes for subsequent consultations. However, as the number of consultations per patient was lower than we had expected, the total time spent on face-to-face contact was not exceeded.

The most unexpected deviation from protocol was that the topic of self-management was not frequently addressed: only in 19% of the patients was this topic discussed. Interviews with the nurses revealed that they considered self-management not suitable for this patient group in this phase, which explains why they did not promote it. According to the nurses, the topic of self-management is more appropriate for a later phase, and can be better practised during more extensive interventions. Indeed, most previous studies on self-management have been performed in chronic conditions [19], and the two effective self-management interventions for survivors of cardiac arrest were much more elaborate and consisted of eight and eleven sessions respectively [6,7]. We therefore conclude that it is not feasible to address self-management properly in the current intervention and propose to eliminate it as one of the obligatory elements.

### Limitations of the process evaluation

A risk in evaluation studies is that participants may tend to give socially desirable answers. We tried to prevent this by providing anonymous evaluations forms to patients and caregivers, and by sending out these forms after the intervention had finished.

The interviews and evaluation forms for the nurses were not anonymous. To limit the risk that the nurses would provide socially desirable answers, the interviews were administered by a researcher who was not involved in the trial. Moreover, the nurses did not have any formal or personal relationships with the researchers before or after the trial.

### Suggestions for implementation

This process evaluation has shown that the intervention is sufficiently feasible. However, there can be some tension between ‘a flexible intervention’ and guaranteeing that certain content is sufficiently addressed. The advantage of a flexible intervention is that it can be tailored to the actual needs of the participants and, in case there are no problems or questions, the number of consultations remains limited, which will positively affect the cost-effectiveness. We have noticed structural differences between the nurses with regard to their performance of the intervention, and believe that the feasibility and reproducibility of the intervention can be improved on some aspects by making several of the optional elements of the intervention more obligatory.

First of all, we suggest offering at least two consultations to all patients. When not taking into account the nurse who had only consulted one patient, the two nurses who consulted patients most frequently also received the highest grades for the intervention and their performance. This suggests that conducting several consultations improves the perceived quality of the intervention.

Secondly, we advise administering a formal screening instrument to all patients. The nurses that used the Checklist Cognition and Emotion were very positive about it, and previous research in stroke patients also showed that formal screening significantly contributed to the detection of cognitive and emotional problems [21]. We think that making such a screening instrument obligatory will secure that ‘screening for cognitive and emotional problems’ is effectuated in all patients.

During the trial, most consultations were conducted as home visits. This seemed to be appreciated by the patients, but also demonstrated the disadvantage that it is more time consuming for the nurse. We recommend that home visits should remain possible but suggest combining the consultation with the nurse with regular out-patient consultations with the cardiologist. As such, the extra effort for the patient is limited while it can improve efficiency for the nurse.

## Conclusions

In conclusion, the intervention ‘Stand still …, and move on’ is a promising intervention, which seems to be feasible and was positively evaluated by patients, caregivers and nurses. Although the intervention was performed according to protocol on most aspects, we have reported a few deviations and we have made recommendations how to address this in future implementation. If the intervention turns out to be (cost-) effective we recommend implementation in regular health care.

## Competing interests

The authors declare that they have no competing interests.

## Authors’ contributions

VM was the principle investigator on this project. She was involved in the design of the intervention, the design of the process evaluation, data collection, data analysis and wrote the drafts of the manuscript. JvH contributed to the design of the process evaluation, performed the semi-structured interviews and supervised data analysis. DW was involved in the design of the intervention and the ALASCA trial. JV was the consultant in rehabilitation medicine that the nurses could contact throughout the intervention period. CvH and JV supervised the project and were involved in the design of the intervention, the design of the process evaluation and interpretation of the results. All authors read, critically reviewed and approved the final manuscript.

## Pre-publication history

The pre-publication history for this paper can be accessed here:

http://www.biomedcentral.com/1472-6963/14/34/prepub
